# Risk prediction models for acute kidney injury in adults: An overview of systematic reviews

**DOI:** 10.1371/journal.pone.0248899

**Published:** 2021-04-01

**Authors:** Paulien Van Acker, Wim Van Biesen, Evi V. Nagler, Muguet Koobasi, Nic Veys, Jill Vanmassenhove

**Affiliations:** Department of internal Medicine, Renal Division, Ghent University Hospital, Ghent, Belgium; University of Catanzaro, ITALY

## Abstract

**Background:**

The incidence of Acute Kidney Injury (AKI) and its human and economic cost is increasing steadily. One way to reduce the burden associated with AKI is to prevent the event altogether. An important step in prevention lies in AKI risk prediction. Due to the increasing number of available risk prediction models (RPMs) clinicians need to be able to rely on systematic reviews (SRs) to provide an objective assessment on which RPM can be used in a specific setting. Our aim was to assess the quality of SRs of RPMs in AKI.

**Methods:**

The protocol for this overview was registered in PROSPERO. MEDLINE and Embase were searched for SRs of RPMs of AKI in any setting from 2003 till August 2020. We used the ROBIS tool to assess the methodological quality of the retrieved SRs.

**Results:**

Eight SRs were retrieved. All studies were assessed as being at high risk for bias using the ROBIS tool. Eight reviews had a high risk of bias in study eligibility criteria (domain 1), five for study identification and selection (domain 2), seven for data collection and appraisal (domain 3) and seven for synthesis and findings (domain 4). Five reviews were scored at high risk of bias across all four domains. Risk of bias assessment with a formal risk of bias tool was only performed in five reviews. Primary studies were heterogeneous and used a wide range of AKI definitions. Only 19 unique RPM were externally validated, of which 11 had only 1 external validation report.

**Conclusion:**

The methodological quality of SRs of RPMs of AKI is inconsistent. Most SRs lack a formal risk of bias assessment. SRs ought to adhere to certain standard quality criteria so that clinicians can rely on them to select a RPM for use in an individual patient.

**Trial registration:**

PROSPERO registration number is CRD 42020204236, available at https://www.crd.york.ac.uk/prospero/display_record.php?RecordID=204236.

## Introduction

The use of more complex technical procedures and interventions in an overall elderly population burdened with comorbidities, has led to a global increase in the incidence of acute kidney injury (AKI) [1]. People who develop AKI during their stay in hospital are about twice as likely to die than those who don’t. Around a third of patients hospitalized with AKI will retain a degree of kidney damage, and their risk of progressive chronic kidney disease will be up to twice as high [2–4]. Often, AKI is not readily detected, even when its development is predictable and avoidable [5]. As it has proved difficult to find effective interventions for reversing established AKI, the focus has gradually shifted from treatment to early diagnosis and prevention of AKI [6].

Key to preventing AKI is the ability to predict AKI *before* it actually occurs. To that end, many mathematical models have been developed using different combinations of laboratory and clinical variables, comorbidities and demographics. AKI is a heterogeneous disease, requiring distinct risk prediction models (RPMs), adapted to the setting of implementation. So many have been published however, that it has become difficult to identify which ones can be reliably used for predicting individual risk in any specific setting.

Systematic reviews (SRs) of RPMs for AKI aim to comprehensively search for and critically appraise RPMs for their accuracy, discriminatory properties and external validity. Such SRs should help us to identify reliable RPMs, which can be implemented in daily practice and improve patient outcome. But for such SRs to draw reliable and transparent conclusions, they must adhere to certain methodological standards [7–9]. Increasingly, methods consortia have issued guidance and checklists on how to conceive and conduct SRs of RPMs [10]. Adherence to such guidance should ensure all relevant studies are captured and a formal assessment of the risk of bias in the included studies, allowing critical appraisal of the evidence, occurred [7,8,11–13]. SRs of RPMs that do not comply with these standards may not only fail to improve but even harm patient outcome.

We aimed to provide an overview of SRs of RPMs for AKI in adults across different settings and critically appraised these reviews to explore if they could be reliably used for selecting AKI RPMs for large scale routine clinical application.

## Methods

We followed the recommendations from Cochrane to conduct this overview of SRs (Cochrane Handbook for Systematic Reviews of Interventions version 6.1 (updated September 2020). Cochrane, 2020. Available from www.training.cochrane.org/handbook) [14].

### Criteria for selection of studies

We included SRs of RPMs for AKI in adults in any setting. Reviews including only children were excluded. We defined a SR of RPMs for AKI as any study that identified itself as such and had aimed to conduct a comprehensive search for primary studies with the aim of providing an up-to-date summary of the state of research knowledge on AKI risk prediction. We did not restrict based on the study design of the primary studies included in the review. Also, given our goal to assess the risk of bias of the SR process, we placed no restriction on methods for critically appraising the included studies.

The protocol for this review was registered at PROSPERO (CRD 42020204236) available at https://www.crd.york.ac.uk/prospero/display_record.php?RecordID=204236.

We reported according to the Preferred Reporting Items for SRs and Meta-Analyses guidelines (PRISMA) [12] and followed the guidelines for Meta-Analyses and SRs of Observational Studies (MOOSE) [13] and consulted the Checklist for critical Appraisal and data extraction for SRs of prediction Modelling Studies (CHARMS) during data extraction [7].

### Search methods

We searched MEDLINE (2003 to August Week 1, 2020) and Embase (2003 to August 2020) combining vocabulary terms and text words for AKI and prognostic studies. We did not apply any methodological filter for identifying SRs because we wanted to avoid missing reviews that had been misclassified and we wanted to screen for additional primary studies on RPMs for AKI for further research purposes. We searched both databases from 2003 onwards as the AKI definition became more standardized with the advent of RIFLE (and later AKIN and KDIGO) from that moment on [15–17]. The search was restricted to English language publications for reasons of feasibility. The full search strategy is available as supplemental material Item S1 File and via the PROSPERO website. References of relevant reviews were screened to identify additional reports. Two authors (PVA and JV) independently screened titles and abstracts and subsequently reviewed full texts for inclusion according to the preset inclusion criteria. Discrepancies were resolved by discussion with a third author (WVB).

### Data collection process and data items

We developed a draft data extraction form which was piloted and modified as necessary. The extracted data included the following: start/stop search period, publication date, population/setting, number of included patients, AKI definition and timing of assessment, AKI incidence, number of included reports, number of included RPMs, number of internally validated RPMs, number of externally validated RPMs, ROBIS domains, conflict of interest/funding.

PVA and JV extracted all data using the standardized data extraction form, any discrepancies were resolved by consensus.

It was beyond the scope of this overview to judge the accuracy of individual RPMs included in the reviews. However, we felt informed assessment of a review’s risk of bias required description of the AKI definitions researchers used and whether these models were internally or externally validated.

### Risk of bias in systematic reviews of risk prediction models

The risk of bias in the included reviews was assessed according to the Risk of Bias in Systematic Reviews (ROBIS) tool [18], in line with recommendations made by the Cochrane handbook [10]. The tool involves three phases. (S1 Table) In summary, the first phase assesses relevance; an optional assessment not conducted within this overview as the focus of interest was the methodological reliability of the SR. The second phase identifies concerns with the SR process. This phase covers four domains as possible sources of bias in the SR process: study eligibility criteria, identification and selection of studies, data collection and study appraisal, and synthesis and findings. The third and final phase judges overall risk of bias for the SR (low, high, unclear).

In view of the expected clinical heterogeneity (low similarity in studies, considering different settings with different case mix and variability in the outcome definition) we did not perform a quantitative analysis but opted for a qualitative analysis.

### Synthesis of systematic review of risk prediction model findings

Given we expected the SRs to cover distinct populations, and distinct RPMs (with or without one or multiple external validation studies), meaningful statistical meta-analysis was not expected to have occurred in the included SRs. Hence, we decided to narratively synthetize the included SRs according to the population and setting, the number of included reports and participants, the number of AKI definitions, the search dates and the risk of bias tool used. We also narratively synthetized information on the number of internal and external validation studies and reports within each SR.

## Results

### Retrieval of studies

**Fig 1:**
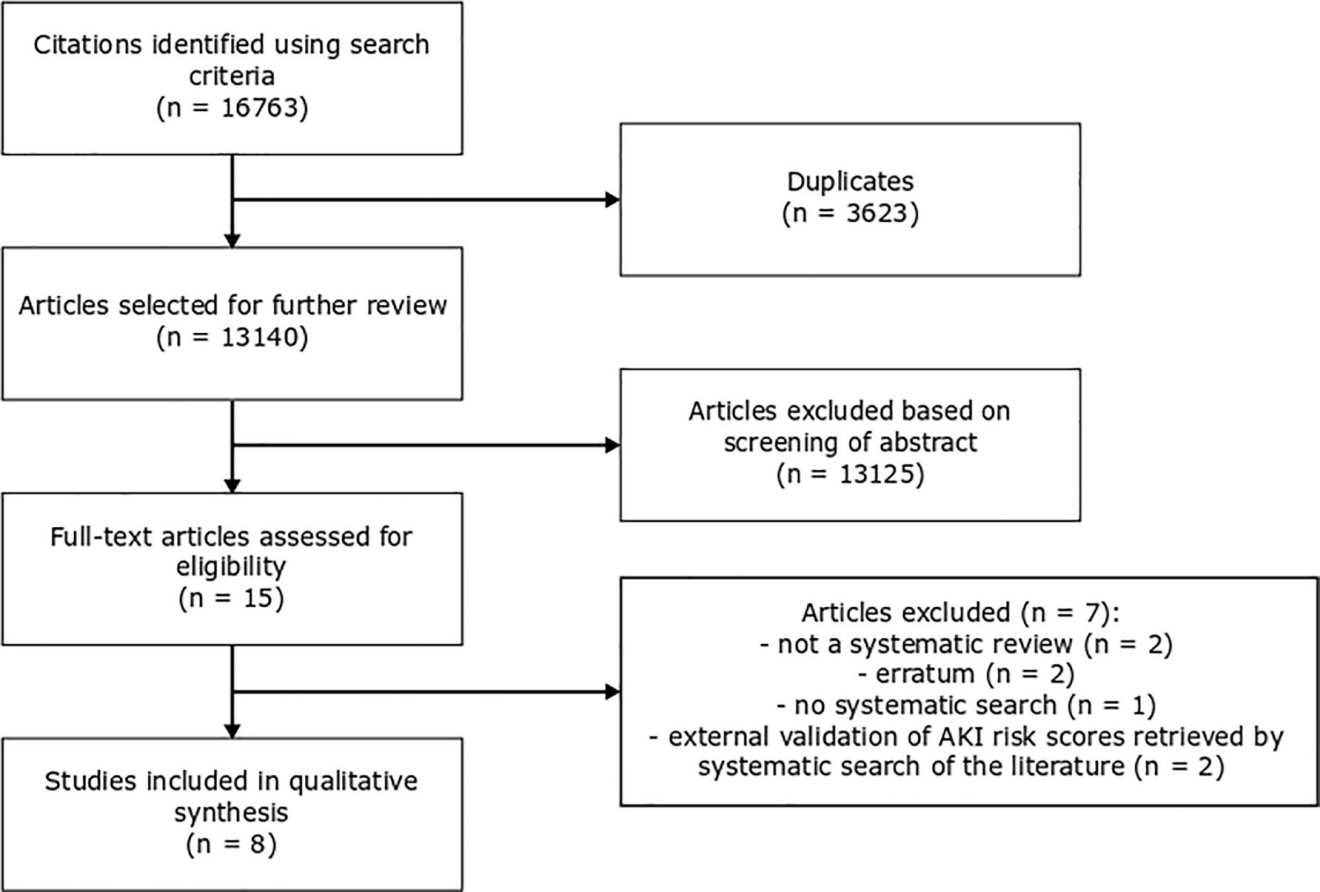
Flow chart of study selection.

A total of 16763 citations (i.e. 5165 in MEDLINE and 11598 in Embase) were identified. After title and abstract screening, 3623 duplicates were removed. Fifteen articles were considered for full text review. Following full text review, eight studies were included in the overview. Screening references of the included reviews did not reveal additional citations for inclusion. A flow chart of study selection is provided in Fig 1.

### Description of the included systematic reviews

Review characteristics are summarized in Tables 1 and 2. Eight author teams each conducted a SR covering RPMs in a distinct setting: AKI after cardiac surgery [19], AKI after major non-cardiac surgery [20], AKI in general hospital populations [21], contrast-associated AKI after a diagnostic or interventional procedure with iodinated radiocontrast [22], contrast-associated AKI after angiography or angioplasty [23], AKI in the context of rhabdomyolysis [24], AKI after liver transplantation [25], and AKI in a general intensive care unit (ICU) [26]. The number of included studies with internal validation varied from four [25] to 68 [23] covering five to 70 RPMs in 2171 and 1.272.712*** study participants. (Tables 1 and 2, ***: see footnote Table 1).

**Table 1:** Review characteristics.

Author Journal Year	Population/setting	Included reports	Included patients*	Number of acute kidney injury definitions**	Databases searched	Dates search started and stopped	Risk of bias tool
Allen*** Canadian Journal of Cardiology **2017 [23]**	Coronary angiography or angioplasty	75	1.272.712	24	MEDLINE Embase	1950 until 31 March 2016 1980 until 31 March 2016	Checklist according to CHARMS and TRIPOD
**Caragata** **Anaesth Intensive Care** **2016 [25]**	Post liver transplantation	7	2.171	6	MEDLINE	Prior to May 2015 until May 2015	No risk of bias assessment
**Hodgson** **BMJ Open** **2017 [21]**	Hospital acquired acute kidney injury	13	349.825	9	MEDLINE Embase Web of Science	Inception until November 2016	PROBAST TRIPOD score
**Huang** **Revista Brasileira de Terapia Intensiva** **2020 [26]**	General ICU patients	5	38.071	5	MEDLINE	1 January 2012 until 5 June 2019	No risk of bias assessment
**Huen** **The Annals of Thoracic Surgery** **2012 [19]**	Post cardiac surgery	15	701.761	5	MEDLINE Web of Science/knowledge Scopus	1950 until May 2011	Predefined questions to assess the methodological quality were developed by the authors
**Safari** **Iranian Journal of Kidney Diseases** **2016 [24]**	Rhabdomyolysis-induced acute kidney injury	6	4.962	No statement	MEDLINE Embase Cochrane Library Scopus Google Scholar	“without any time limitation”	No risk of bias assessment
**Silver** **BMJ** **2015 [22]**	Diagnostic or interventional procedure that used conventional, iodinated radiocontrast	16	119.461	9	MEDLINE Embase CINAHL	1946 until 9 March 2015 1947 until week 10 2015 1993 until March 2015	Risk of bias assessment based on modified criteria by Hayden [27]
**Wilson** **Nephrology Dialysis Transplantation** **2016 [20]**	Major non-cardiac surgery	6	78.331	5	MEDLINE Embase BIOSIS Previews Web of Science	Inception until 30 June 2014	Quality assessment based on TRIPOD

Abbreviations: TRIPOD: Transparent Reporting of a multivariable prediction model for Individual Prognosis Or Diagnosis, CHARMS: Checklist for critical Appraisal and data extraction for systematic Reviews of prediction Modelling Studies, PROBAST: Prediction model Risk of Bias Assessment Tool.

* Total number based on the numbers provided by the authors of the review, for further elaboration and reporting of potentially missing numbers see supplementary S3 Table.

** For further elaboration on the AKI definitions used and the number of AKI events, see supplementary S3 Table.

*** For Allen et al. the provided number of included patients refers to the participants included in the 30 models that provided sufficient information to obtain individual risk estimates. The number of participants for all 75 reports is not provided.

**Table 2:** Number of reports and models with both internal and/or external validation, included in the systematic reviews.

Author	Population/setting	Included reports	Included risk prediction models	Internal validation reports	Risk prediction models with internal validation	External validation reports	Risk prediction models with external validation
**Allen#**	Coronary angiography or angioplasty	75	70 (of which 30 provided a risk score and were discussed)	68	70	19	9
Caragata**	Post liver transplantation	7	9	4	5	0	0
**Hodgson§** **	Hospital acquired AKI	13	11	9	9	6	5
**Huang**	General ICU patients	5	8	5	8	1*	1*
**Huen**	Post cardiac surgery	15	7	7	7	9	4
**Safari**	Rhabdomyolysis-induced AKI	6	7	0	0	0	0
**Silver#**	Diagnostic or interventional procedure that used conventional, iodinated radiocontrast	16	12	10	10	8	6
**Wilson§**	Major non-cardiac surgery	6	7	6	7	0	0

* Huang et al considered the risk prediction model by Flechet et al. as externally validated. However, the model was validated in an independent split sample of the original data which should thus be considered as an internal validation report.

# There is an overlap in included risk prediction models between Silver et al. and Allen et al.

§There is an overlap in included risk prediction models between Hodgson et al. and Wilson et al.

** There is an overlap in included risk prediction models between Hodgson et al. and Caragata et al.

All reviews were published between 2012 and 2020. Only 3 of the SRs published a statement on conflict of interest and on funding of the SR. None of the SRs reported if the included primary studies had a statement on conflict of interest or funding [20,22,23].

### Risk of bias in the included systematic reviews—using the ROBIS tool

**Fig 2:**
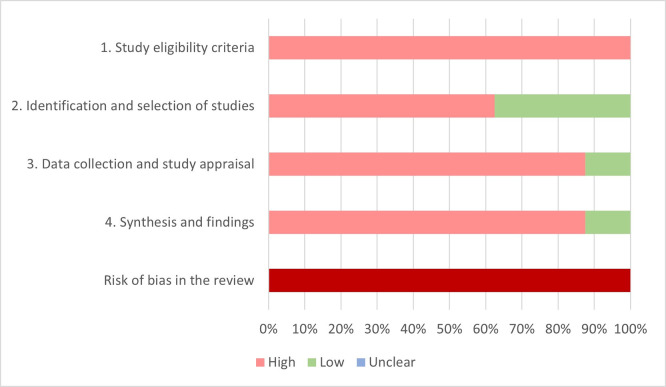
Summary of ROBIS assessment. Percentages are based on number of included systematic reviews (100% = 8 included systematic reviews).

**Fig 3:**
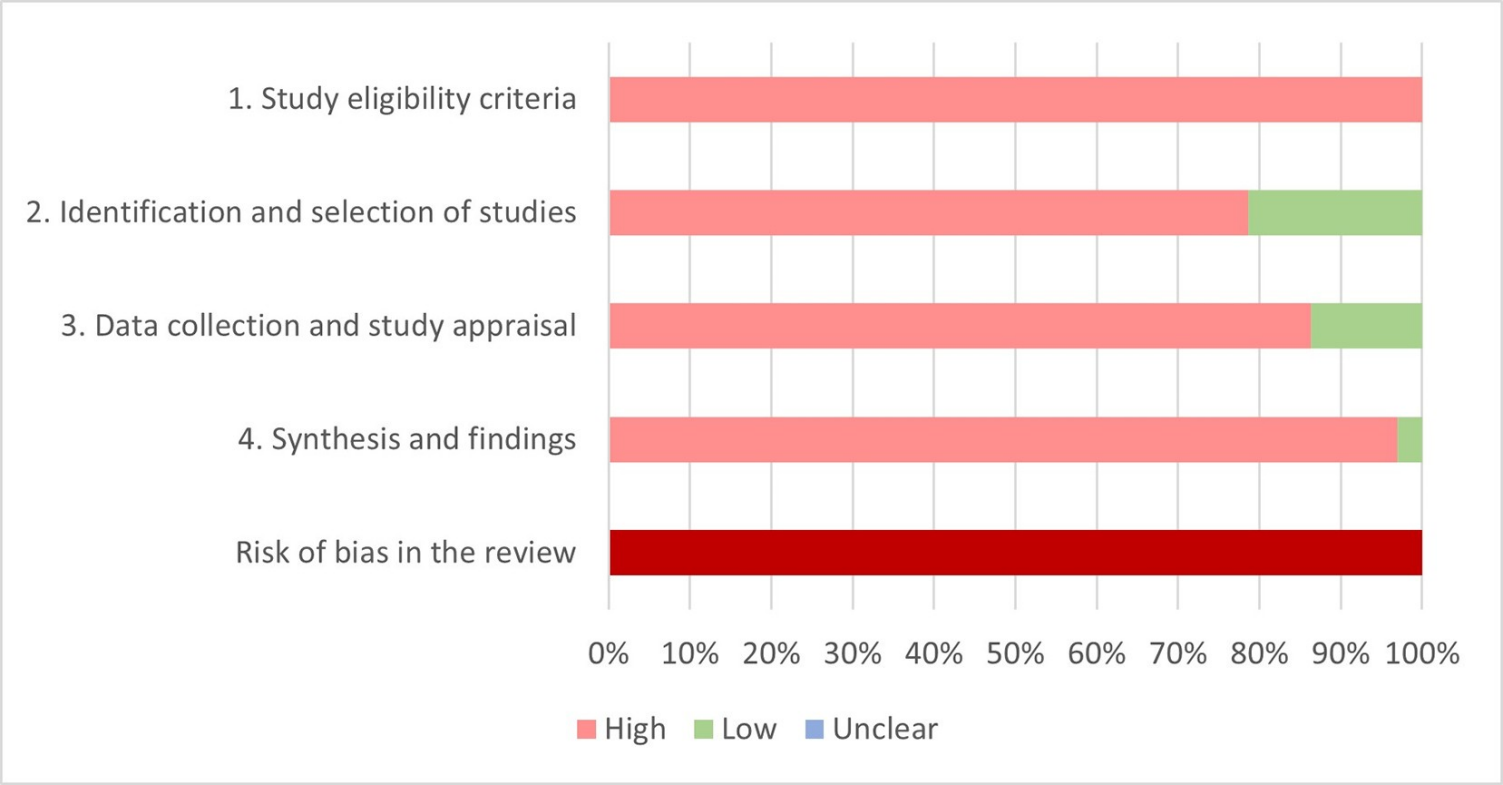
Summary of ROBIS assessment. Percentages are based on number of included patients in the different reviews (100% = 2.567.474*^,^** included patients) * For Hodgson et al. the number of participants only relates to the development studies since values for the number of patients included in the external validation studies are not provided in the systematic review. ** For Allen et al. the number of included patients relates to the total number of patients included in the 30 models that provided sufficient information to obtain individual risk estimates. The number of participants for all 75 reports is not provided.

Figs 2 and 3 show the risk of bias judgment for each domain separately and across domains as a proportion of the included reviews and number of patients. Individual ratings for underlying signaling questions are detailed in Table 3 and S2 Table. All reviews were estimated to be at high risk of bias overall.

**Table 3:** Summary of ROBIS assessment.

	Phase 2																					Phase 3
	1 Study eligibility criteria					2 Identification and selection of studies					3 Data collection and study appraisal					4 Synthesis and findings						Risk of bias
	1.1	1.2	1.3	1.4	1.5	2.1	2.2	2.3	2.4	2.5	3.1	3.2	3.3	3.4	3.5	4.1	4.2	4.3	4.4	4.5	4.6	
Allen	PY	Y	N	Y	N	Y	Y	Y	N	Y	Y	N	Y	Y	PY	PN	N	PN	N	N	N	High
	High					High					High					High						
Caragata	N	Y	N	N	N	N	Y	N	N	PN	PN	N	Y	N	N	Y	NI	PN	N	N	N	High
	High					High					High					High						
Hodgson	PY	Y	Y	Y	N	Y	Y	Y	Y	Y	Y	Y	Y	Y	Y	N	Y	Y	Y	Y	Y	High
	High					Low					Low					High						
Huang	N	Y	Y	PY	Y	N	Y	N	N	PN	PN	N	Y	N	N	Y	NI	Y	Y	N	N	High
	High					High					High					High						
Huen	N	Y	N	N	N	Y	Y	N	Y	PN	PN	N	Y	PY	PN	N	NI	Y	Y	Y	N	High
	High					High					High					High						
Safari	N	N	N	N	PY	Y	PY	N	PY	Y	PY	N	N	N	PY	Y	NI	PN	Y	N	N	High
	High					High					High					High						
Silver	N	Y	N	PN	N	Y	Y	Y	PY	Y	NI	N	Y	Y	Y	N	NI	Y	Y	Y	Y	High
	High					Low					High					High						
Wilson	PY	Y	N	Y	Y	Y	Y	Y	Y	Y	Y	N	Y	Y	NI	Y	PY	Y	Y	Y	Y	High
	High					Low					High					Low						

Abbreviations: N = No; NI = no information; PN = probably no; PY = probably yes; Y = yes.

Only two reviews mention a predesigned protocol but without providing a link to an accessible version [20,23]. One review group elaborates on predefined eligibility criteria provided in the supplementary material. All reviews were considered as having high risk of bias in the study eligibility criteria (domain 1) because they lacked unambiguous eligibility criteria (all except Hodgson et al. [21] and Huang et al. [26]), the study population was only vaguely described, a language restriction was applied [22,23], an inappropriate restriction on the outcome definition was applied [22], unpublished conference abstracts had been excluded [19,21,22] or studies had been excluded based on sample size or quality [24].

All but two reviews were judged to be at ‘high risk of bias’ for the identification and selection of studies (domain 2) [19,23–26]. Some did not provide a reproducible full search strategy [19,24–26], or applied inappropriate or ambiguous restrictions based on time [23,24], languag e [25,26] or publication format [26].

Only one SR obtained a low risk of bias for data collection and study appraisal (domain 3) [21]. Reviews judged at high risk of bias either provided incomplete information on characteristics of the included primary studies [19,20,22–26] or did not provide a formal risk of bias assessment [24–26]. Information on included variables was limited in the majority of reviews and only two mention the rationale behind the choice of predictor variables and the initial number of variables considered [21,23]. None of the reviews report if the original studies covered the methods for assessment of the predictor variables and the timing of their assessment. Several reviews do not mention the inclusion and exclusion criteria of the original studies [22–24,26]. Only one clearly provided the in- and exclusion criteria of the included RPMs [21]. One only provided the exclusion criteria [25]. Both Huen et al. [19] and Wilson et al. [20] checked whether the authors of the included RPMs provided clear in- and exclusion criteria but did not provide the actual criteria in the text or as supplementary material. Only one review explicitly mentioned that study selection, data extraction and risk of bias assessment were done by two independent reviewers [21].

Several reviews omitted some studies and RPMs in their synthesis [19,21–23]. Three reviews did not assess the risk of bias of the included external validation studies [19,21,22]. Most reviews opted for a qualitative synthesis because of important heterogeneity; two conducted a partly quantitative analysis [23,25]. Overall, only one was scored as low risk of bias in domain 4—synthesis and findings [20].

### Description of the risk prediction models included in the systematic reviews

Of the 75 included studies by Allen et al. [23] only 30 models provided an individual risk prediction tool and are considered.

We identified 75 unique RPMs. Only 19 RPMs were externally validated. Of these, 11 RPMs were validated in a single validation study. One model was validated in 14 validation reports (Mehran et al. 2004, contrast-associated AKI), one model in six (Thakar et al. 2005, cardiac surgery, Cleveland Clinic Score), two models in five (Chertow et al.1997, cardiac surgery, CICSS; Bartholomew et al. 2004, contrast associated-AKI), one model in four (Wijeysundera et al. 2007, cardiac surgery), two models in three (Mehta et al. 2006, cardiac surgery, STS; Tziakas et al. 2013, contrast associated-AKI) and one model in two (Forman et al. 2004, heart failure).

### Description of the outcome being predicted: AKI

Although on a semantic level the same outcome definition (Acute Kidney Injury, AKI) was *seemingly* used in all studies, its actual interpretation varied strongly across the originally included papers and across the SRs. We identified five [19,20,26] to 24 [23] different AKI definitions for seven [19,20], eight [26] and 30 models [23] respectively (see Tables 1 and 2). All AKI definitions that were applied in the original RPM studies and the AKI event rates are provided in the supplementary material (S3 Table), if reported by the authors of the SR. Of the 73 reports in which an AKI definition was provided, 53 different AKI definitions were found. Most original studies defined AKI based on the serum creatinine (sCr) criterion only. Only five original studies across all SRs included the urinary output criterion in their definition (Nyman-2008, Chiofolo-2019, Deng-2017, Slankamenac-2009 and Slankamenac-2013). None of the included reviews considered criteria for AKI other than based on sCr and/or urinary output. The most frequently used AKI definition is based on a sCr increase of at least 0.5mg/dl or an increase of 25% above baseline. However, even across studies using this definition, the timing of assessment differs. For the majority of the included external validation studies, the AKI definition and/or event rate is not or incompletely provided. The outcome definition in the external validation studies is expected to be identical to the definition used in the original development study. However, in those reviews that (partially) provide this information, the outcome definition sometimes differs from the original definition. Allen et al. [23] found that only five of the 19 external validation studies used the same definition as was used in the original development study. Two external validation reports in Silver et al. [22] use a different definition to the development study (Tziakas et al.2013 validating Bartholomew et al. and Gao et al. 2014 validating Mehran et al.). The external validation study by Wang et al. included in Hodgson et al. [21], also used a different definition than the original development study.

## Discussion

Currently, no effective interventions for reversing established AKI exist. Prevention is therefore key to improving patient outcome. Models that can reliably predict who is at risk for AKI, could help identify those in need of specialized care, guide decision making and avoid additional renal insults. But RPMs can only improve patient outcome if their results are robust and accurate.

A SR is considered the highest level of evidence. That can only be true if one can rely on its findings. We conducted an overview of SRs of RPMs for predicting AKI and found eight reviews covering eight distinct settings: AKI after cardiac surgery [19], AKI after major non-cardiac surgery [20], AKI in general hospital populations [21], contrast-associated AKI after a diagnostic or interventional procedure with iodinated radiocontrast [22], contrast-associated AKI after angiography or angioplasty [23], AKI in the context of rhabdomyolysis [24], AKI after liver transplantation [25], and AKI in a general intensive care unit (ICU) [26]. All reviews were considered at high risk of bias. Although a formal critical appraisal of the included primary studies is an essential step in any SR process, this was not done in 3 of the 8 reviews [24–26].

During the last two decades, many RPMs have been published. In the reviews identified by this overview, 75 unique original AKI RPMs were presented. To the end-user, it would be nearly impossible to independently assess the reliability of every risk prediction modelling study. Clinicians need to be able to rely on SRs, allowing them to select a RPM that is applicable in the context of their individual patient. As in other populations, finding robust RPMs that can be personalized according to the setting is a major challenge [28].

Even if a RPM performs well at predicting AKI, this will only lead to improved patient outcome if the action taken based on that prediction actually makes a difference. Only three reviews highlighted the importance of this topic and none identified a RPM assessing the impact of its implementation in clinical practice, on patient outcome [20,21,25].

In recent years, emphasis on methodological rigor in systematic reviewing has increased and several methodological tools have facilitated the adherence to predefined standards. In 2016, the ROBIS tool was developed to specifically assess the risk of bias in SRs [18]. Phase 2 of the ROBIS tool covers four domains assessing study eligibility criteria (domain 1), identification and selection of studies (domain 2), data collection and study appraisal (domain 3) and synthesis and findings (domain 4).

All eight reviews included in this overview were scored at ‘high risk’ for bias when assessing the study eligibility criteria [19–26]. These criteria must clearly identify the scope of the review. The review question and eligibility criteria allow the user to judge if a SR is appropriate for risk prediction in the setting and the patient population it is intended for.

Due to the heterogeneous nature of AKI with various underlying pathophysiological pathways, it is likely that the choice of a RPM will need to be adapted to the setting of implementation.

Essential details in the eligibility criteria were often lacking. The choice and rationale of which study design could represent the basis for RPM building is essential and yet hardly ever discussed. RPMs developed based on information retrieved from prospective observational cohorts are likely more reliable compared to models developed based on retrospective data.

To increase consistency and avoid errors while assessing the individual studies in a SR, all steps of the review process, from study selection to data extraction and risk of bias assessment, are best conducted by two reviewers independently of each other with rules on handling discrepancies. Only one review unambiguously stated they had done so [21].

Incomplete reporting of primary study characteristics and results hamper valid contextualization and conclusions. Information on the exact setting, the number of participants, the outcome definition and timing of assessment, the number of events, demographics and comorbidities, how authors dealt with missing data was only sufficiently reported in one review [21]. Information on how patients lost to follow-up were handled was lacking for all SRs.

The performance of a model is subject to the methods that were used to build it. All but three SRs [22,24,25] provided information on model building. Failing to report on the initial number of considered predictor variables makes it impossible to calculate the event rate/predictor ratio. If the number of events in relation to the considered regression coefficients is fewer than 10, the risk for overfitting increases substantially. However, only one review [21] reported clearly on the initial number of variables that were considered in each development model. The model performance of individual risk prediction models [28] can be assessed by evaluating calibration or predicted vs observed probability e.g. by using calibration plot, calibration slope, or Hosmer-Lemeshow test, and by evaluating discrimination or the ability to distinguish patients with AKI from those who do not have AKI, which is usually done by providing the c statistic with a confidence interval. None of the SRs consistently provided these measures of performance for all of the included internal and external validation reports.

External validation in a population different from that used to develop the model is required to learn if a model can be implemented outside the population for which it was originally developed. It thus assesses the generalizability of the model [29]. All SRs included in this review discuss if models were internally and externally validated but often lack crucial information on whether the external validation models use the same regression coefficients and number of predictors as in the development study. None of the reviews consistently provide information on the AKI definition used in all the included external validation studies or whether this AKI definition differs from the original development study.

The wide range of AKI definitions is a substantial source of heterogeneity between the different RPMs. A staggering 53 different definitions for AKI were retrieved in the 73 reports that provided an AKI definition. This is remarkable considering there is a clear incentive for the standardization of the AKI definition since 2004 [15–17]. It underpins the problems clinicians are facing when they want to implement a RPM but often are confronted with different interpretations of the ‘AKI construct’ in the prediction algorithm. A model performing well predicting AKI defined based on the sCr criterion will not necessarily perform well if the AKI construct is changed and AKI is defined based on both the sCr and UO criterion. As in other settings, it also underlines the importance of joining forces within the nephrology community [30], gathering data in large international databases, using a standardized AKI definition in the prediction algorithm and involving different stakeholders in order to build reliable RPMs across different settings and populations.

Until recently, there was no formal tool for risk of bias assessment in RPMs. In 2017 the PROBAST (Prediction model Risk Of Bias Assessment Tool) tool was specifically developed for that purpose [8]. Until the advent of PROBAST, authors had to adapt existing tools that were originally developed for other study designs or use the TRIPOD (Transparent Reporting of a multivariable prediction model for Individual Prognosis or Diagnosis) checklist to formulate important items for risk of bias assessment in RPMs. Only one review [21] used the PROBAST tool (before its publication), six reviews were published before 2017 and did not have this tool at their disposal. Finally, one review published in 2020 did not perform any risk of bias assessment [26].

We recommend future SRs in the field to use the CHARMS (Critical Appraisal and Data Extraction for Systematic Reviews of Prediction Modelling Studies) checklist [7] to ensure all relevant data from the individual studies are extracted; apply the PROBAST tool for individual risk of bias assessment for each development and external validation study; and follow MOOSE (Meta-analyses of Observational Studies in Epidemiology) guidelines to ensure adequate reporting [13]. We suggest authors developing RPMs consult the TRIPOD checklist [11] beforehand so that all relevant items are considered in the development of the model and its reporting.

## Limitations and strengths

The strength of this review is that we performed an extensive literature search and that both study selection, data extraction and risk of bias assessment were done by two reviewers independently of one another. We uncovered several shortcomings that hinder progression in the field of AKI prediction. Acknowledging and tackling these obstacles could help moving in the right direction and improve patient outcome. Considering the expected large number of heterogeneous RPMs across different AKI settings, we decided to limit the search to studies published from 2003 onwards. We chose this starting point because the RIFLE criteria were published in 2004 and a more homogenous standardized AKI definition was expected from then on. In addition, it is reasonable to assume that health care has changed substantially over the last 20 years, and that RPMs developed before 2000 are in any case unlikely generalizable to the current setting. We limited the search to papers published in English and did not search for unpublished studies or grey literature. However, by doing so it seems unlikely that we would have missed additional reviews of high methodological quality or that the conclusion of this review would have changed.

## Conclusion

There has been a shift in focus towards prevention of AKI from trying to find treatments for AKI or mitigating its course. The role of risk prediction modelling in prevention is potentially crucial and the advent of performant electronic health records allowing automated prediction of risk for AKI can mean a leap forward. However, its potential impact depends on the quality and reliability of the RPM that is implemented. SRs of RPMs are considered the most reliable source of evidence to make a well-founded choice on which RPM is most suited for a particular context. However, across different settings of AKI, SRs of RPMs show inconsistent quality. Individual RPM studies and SRs of RPMs that adhere to good methodological standards have the best opportunity to positively impact patient outcome, and benefit guideline development and health policy.

## Supporting information

S1 Checklist PRISMA 2009 checklist. (DOC)

S1 Table Summary of phase 2, phase 3, and signaling questions of the ROBIS tool. (PDF)

S2 Table ROBIS assessment of the included systematic reviews with reasoning. (PDF)

S3 Table AKI definition, number of participants and number of AKI events in the included risk prediction models across the reviews. (PDF)

S1 File Search strategy. (PDF)
