# Supplementary material for: Risk prediction models for acute kidney injury in adults: An overview of systematic reviews
Source: PLoS One. 2021 Apr 1;16(4):e0248899. doi: 10.1371/journal.pone.0248899 (PMC8016311; doi:10.1371/journal.pone.0248899)
Supplement: S1 Table — (PDF) [file pone.0248899.s002.pdf]

Supplementary material: Summary of phase 2 ROBIS domains, phase 3, and signaling questions.

	Phase 2				Phase 3
	**1. Study eligibility criteria**	**2. Identification and selection of studies**	**3. Data collection and study appraisal**	**4. Synthesis and findings**	**Risk of bias in the review**
Signaling questions	1.1 Did the review adhere to predefined objectives and eligibility criteria?	2.1 Did the search include an appropriate range of databases/ electronic sources for published and unpublished reports?	3.1. Were efforts made to minimize error in data collection?	4.1. Did the synthesis include all studies that it should?	A. Did the interpretation of findings address all of the concerns identified in domains 1 to 4?
	1.2 Were the eligibility criteria appropriate for the review question?	2.2 Were methods additional to database searching used to identify relevant reports?	3.2. Were sufficient study characteristics available for both review authors and readers to be able to interpret the results?	4.2. Were all predefined analyses reported or departures explained?	B. Was the relevance of identified studies to the review's research question appropriately considered?
	1.3 Were eligibility criteria unambiguous?	2.3 Were the terms and structure of the search strategy likely to retrieve as many eligible studies as possible?	3.3. Were all relevant study results collected for use in the synthesis?	4.3. Was the synthesis appropriate given the nature and similarity in the research questions, study designs, and outcomes across included studies?	C. Did the reviewers avoid emphasizing results on the basis of their statistical significance?
	1.4 Were all restrictions in eligibility criteria based on study characteristics appropriate?	2.4 Were restrictions based on date, publication format, or language appropriate?	3.4. Was risk of bias (or methodologic quality) formally assessed using appropriate criteria?	4.4. Was between-study variation minimal or addressed in the synthesis?	
	1.5 Were any restrictions in eligibility criteria based on sources of information appropriate?	2.5 Were efforts made to minimize error in selection of studies?	3.5. Were efforts made to minimize error in risk of bias assessment?	4.5. Were the findings robust, for example, as demonstrated through funnel plot or sensitivity analyses?	
				4.6. Were biases in primary studies minimal or addressed in the synthesis?	
Judgment	Concerns regarding specification of study eligibility criteria	Concerns regarding methods used to identify and/or select studies	Concerns regarding methods used to collect data and appraise studies	Concerns regarding the synthesis	Risk of bias in the review

Whiting P, Savovic J, Higgins JP, Caldwell DM, Reeves BC, Shea B, Davies P, Kleijnen J, Churchill R, group R: ROBIS: A new tool to assess risk of bias in systematic reviews was developed. J Clin Epidemiol 2016, 69:225-234.
