# Supplementary material for: Risk prediction models for acute kidney injury in adults: An overview of systematic reviews
Source: PLoS One. 2021 Apr 1;16(4):e0248899. doi: 10.1371/journal.pone.0248899 (PMC8016311; doi:10.1371/journal.pone.0248899)
Supplement: S2 Table — (PDF) [file pone.0248899.s003.pdf]

Supplementary material: ROBIS assessment of the included systematic reviews with reasoning.

Domain 1: Study eligibility criteria	Review	Rating	Reasoning
1.1 Did the review adhere to pre-defined objectives and eligibility criteria?	Allen et al.	Probably yes	The authors mention the use of a prespecified study protocol but we were unable to retrieve this protocol (no link or reference is provided).
	Caragata et al.	No	There is no prespecified study protocol.
	Hodgson et al.	Probably yes	Prespecified eligibility criteria are available as supplementary material but there is no prespecified study protocol.
	Huang et al.	No	There is no prespecified study protocol.
	Huen et al.	No	There is no prespecified study protocol.
	Safari et al.	No	There is no prespecified study protocol.
	Silver et al.	No	There is no prespecified study protocol.
	Wilson et al.	Probably yes	The authors mention the use of a prespecified study protocol but we were unable to retrieve this protocol (no link or reference is provided).
1.2 Were the eligibility criteria appropriate for the review question?	Allen et al.	Yes	The provided criteria are appropriate for the review question.
	Caragata et al.	Yes	The provided criteria are appropriate for the review question.
	Hodgson et al.	Yes	Authors clearly defined the eligibility criteria which are appropriate for the review question.
	Huang et al.	Yes	The provided criteria are appropriate for the review question.
	Huen et al.	Yes	The provided criteria are appropriate for the review question.
	Safari et al.	No	Authors do not provide a clearly defined review question.
	Silver et al.	Yes	The provided criteria are appropriate for the review question.
	Wilson et al.	Yes	The provided criteria are appropriate for the review question.
1.3 Were eligibility criteria unambiguous?	Allen et al.	No	The population is not clearly defined. Authors do no not explicitly state whether only studies in adults vs children are eligible. Study design is not clearly mentioned and authors state that "any study" looking at risk prediction will be included. This is in contrast with their statement further on declaring that only studies that provide estimates of AKI risk are considered. It is unclear whether authors clearly make a distinction between risk factor finding studies and RPMs for individual risk prediction.

	Caragata et al.	No	The population is not clearly defined. There is no clear description about which study design is acceptable for the eligible studies.
	Hodgson et al.	Yes	Authors provide clear eligibility criteria in the supplementary material.
	Huang et al.	Yes	Authors provide clear eligibility criteria.
	Huen et al.	No	The population is not clearly defined. Authors do no not explicitly state whether only studies in adults vs children are eligible. There is no clear description about which study design is acceptable for the eligible studies.
	Safari et al.	No	The population is not clearly defined. Authors state that all observational studies with a clear definition of rhabdomyolysis and AKI based on standard scales were entered. The context in which rhabdomyolysis might occur can be very broad and authors fail to elaborate on this. It is unclear what is meant by 'standard scales'. Authors do not mention whether they included development studies, validation studies or both (e.g the MESS score was originally developed for a different outcome). Both 'studies that evaluated AKI risk factors in rhabdomyolysis, as well as those assessing the role of urinary dipstick in this regard', were included. It is unclear why authors categorize 'studies investigating urinary dipstick' as a separate entity.
	Silver et al.	No	Authors state that studies providing both development and validation are considered. They do not distinguish between internal and external validation.
	Wilson et al.	No	The population is not clearly defined. Authors do no not explicitly state whether only studies in adults vs children are eligible. It is not clear whether only validated studies were considered for inclusion. The definition of AKI is vague: "any clinical or laboratory based definition of AKI".
1.4 Were all restrictions in eligibility criteria based on study characteristics appropriate?	Allen et al.	Yes	
	Caragata et al.	No	Studies were excluded if only logistic regression was performed without reporting a scoring system that could be used clinically. This suggests that only studies using logistic regression for risk prediction were included.
	Hodgson et al.	Yes	

	Huang et al.	Probably yes	The aim of the study is to evaluate RPMs for daily clinical use. The authors applied restrictions about study quality that seem appropriate. E.g. they excluded models that were not internally or externally validated.
	Huen et al.	No	Studies were excluded if only logistic regression was performed without reporting a scoring system that could be used clinically, suggesting that only studies using logistic regression for risk prediction were included.
	Safari et al.	No	Three studies were excluded because of poor quality but it is unclear what authors define as poor quality. Not considering studies for this reason makes the review prone to bias. Also, two studies were excluded because of small sample size without any further explanation.
	Silver et al.	Probably no	Only studies defining AKI according to the serum creatinine criterion were included. Omitting the urinary output criterion is not conform the guidelines for defining AKI.
	Wilson et al.	Yes	
1.5 Were any restrictions in eligibility criteria based on sources of information appropriate?	Allen et al.	No	Only studies published in English were included.
	Caragata et al.	No	The eligibility criteria are not well described. Articles were selected for inclusion if they described the primary development of a clinical RPM. It seems like studies that only performed an external validation of a RPM were excluded but this remains unclear.
	Hodgson et al.	No	Conference abstracts were excluded.
	Huang et al.	Yes	No restrictions were mentioned.
	Huen et al.	No	Unpublished conference abstracts were excluded
	Safari et al.	Probably yes	No clear restrictions but the eligibility criteria are not well described.
	Silver et al.	No	Only studies in English were included in this review. Unpublished conference abstracts were excluded.
	Wilson et al.	Yes	No restrictions were used.
Domain 2: Identification and selection of studies	Review	Rating	Reasoning
2.1 Did the search include an appropriate range of databases/electronic sources for	Allen et al.	Yes	MEDLINE and EMBASE were searched.
	Caragata et al.	No	Only MEDLINE was searched.
	Hodgson et al.	Yes	MEDLINE, EMBASE and Web of Science were searched.

published and unpublished reports?	Huang et al.	No	Only PubMed was searched.
	Huen et al.	Yes	Web of Science/Knowledge, Scopus and MEDLINE were searched.
	Safari et al.	Yes	MEDLINE, EMBASE, Cochrane Library, Scopus and Google Scholar were searched.
	Silver et al.	Yes	EMBASE, MEDLINE and CINAHL were searched.
	Wilson et al.	Yes	Ovid MEDLINE, EMBASE, BIOSIS Previews and Web of Science were searched.
2.2 Were methods additional to database searching used to identify relevant reports?	Allen et al.	Yes	The reference lists of the identified relevant publications were searched for additional relevant studies. Experts on the topic were contacted.
	Caragata et al.	Yes	The citing articles and references of relevant publications were subsequently reviewed to identify any additional studies.
	Hodgson et al.	Yes	Reference lists from retrieved articles were searched to retrieve additional publications.
	Huang et al.	Yes	Potentially relevant articles identified by other sources and references of the retrieved literature were also included.
	Huen et al.	Yes	References and citing articles of identified publications were searched for additional studies.
	Safari et al.	Probably yes	The reviewers stated that a hand search was done. It is not clear which articles or reference lists were hand searched.
	Silver et al.	Yes	Additionally, the bibliographies of identified articles were reviewed to retrieve other articles.
	Wilson et al.	Yes	The authors manually reviewed bibliographies and citations.
2.3 Were the terms and structure of the search strategy likely to retrieve as many eligible studies as possible?	Allen et al.	Yes	Search strategy is adequate and available in the supplementary material.
	Caragata et al.	No	Only MESH headings and keywords are described. A full search strategy is not available.
	Hodgson et al.	Yes	Search strategy is adequate and available in the supplementary material.
	Huang et al.	No	Only MESH headings are described. A full search strategy is not available.
	Huen et al.	No	Only MESH headings and keywords without boolean operators are described. No full search strategy is available. The number of retrieved citations also seems exceptionally low (n=227).

	Safari et al.	No	The search strategy is not elaborated enough. Text words and MESH headings for AKI are insufficient, e.g. abbreviations such as "AKI", "ARF" etc. which are frequently used, are not included.
	Silver et al.	Yes	Search strategy is adequate and available in the supplementary material.
	Wilson et al.	Yes	Search strategy is adequate.
2.4 Were restrictions based on date, publication format or language appropriate?	Allen et al.	No	It is unclear why EMBASE was not searched from inception.
	Caragata et al.	No	Only publications in English were searched for. It is not clear in what year the search was started. The authors only mention the following term when referring to the start of the search: "prior to May 2015".
	Hodgson et al.	Yes	No restrictions were applied to the search strategy.
	Huang et al.	No	Only papers in English and only papers with full text availability were searched for.
	Huen et al.	Yes	No restrictions were applied to the search strategy.
	Safari et al.	Probably yes	The search was conducted 'without any time or language restrictions'. However, it is unclear over which time frame the search was performed.
	Silver et al.	Probably yes	In the 'Methods' section the reviewers state that the search was done up to March 2015. However in the search strategy available in the supplementary material online the authors used a different time restriction, namely '2013'. This is probably a typing error but it is confusing.
	Wilson et al.	Yes	No restrictions were applied to the search strategy.
2.5 Were efforts made to minimize error in selection of studies?	Allen et al.	Yes	Two reviewers independently screened the list of retrieved studies for inclusion.
	Caragata et al.	Probably no	No information was provided on how the study selection was performed.
	Hodgson et al.	Yes	Two reviewers independently screened the list of retrieved studies for inclusion.
	Huang et al.	Probably no	No information was provided on how the study selection was performed.
	Huen et al.	Probably no	No information was provided on how the study selection was performed.
	Safari et al.	Yes	Two reviewers independently screened the list of retrieved studies for inclusion.
	Silver et al.	Yes	Two reviewers independently screened the list of retrieved studies for inclusion.

	Wilson et al.	Yes	Two reviewers independently screened the list of retrieved studies for inclusion.
Domain 3: Data collection and study appraisal	Review	Rating	Reasoning
3.1 Were efforts made to minimize error in data collection?	Allen et al.	Yes	Two reviewers independently extracted data from the retrieved studies. There is no mention of a predesigned data extraction form.
	Caragata et al.	Probably no	No predefined data extraction form has been used. It is not clear who performed the data extraction.
	Hodgson et al.	Yes	Two reviewers independently extracted data from the retrieved studies. A data extraction form has been used.
	Huang et al.	Probably no	A data extraction form was used, but it was not specified by whom the data extraction was done.
	Huen et al.	Probably no	No predefined data extraction form has been used. It is not clear who performed the data collection.
	Safari et al.	Probably yes	Data were summarized using a checklist based on the MOOSE statement guidelines. It is not clearly stated that this was done by two independent reviewers.
	Silver et al.	No information	The reviewers write in the ‘Data extraction’ section the following: ‘From each study, ‘we’ abstracted data on baseline patient …’ We cannot retrieve whether two reviewers independently extracted the data.
	Wilson et al.	Yes	The authors used a predesigned data extraction form. Two reviewers independently extracted data.
3.2 Were sufficient study characteristics available for both review authors and readers to be able to interpret the results?	Allen et al.	No	Information on important comorbidities and demographics is lacking, especially for the validation studies. There is no information on the number of predictors that were considered at the start compared to the final number of selected variables, and how these were chosen in the first place. It is unclear whether the predictors were measured/assessed in the same way in the validation vs the development studies. The event rate for the external validation studies is not provided. The exact number of predictors included in each model is not provided.

	Caragata et al.	No	There is no information on comorbidities and demographics of the included participants. There is no information on the number of predictors the authors of the primary studies started off with compared to the final number of selected variables and how these were chosen in the first place.
	Hodgson et al.	Yes	Supplementary material provides the necessary study characteristics.
	Huang et al.	No	Information on important comorbidities and demographics is lacking. There is no information on the number of predictors that were considered at the start compared to the final number of selected variables, and how these were chosen in the first place, for most of the RPMs. It is unclear whether the predictors were measured/assessed in the same way in the validation vs the development study.
	Huen et al.	No	The definition of AKI used in the different original papers is not specified. Authors use the terms 'AKI-D' and 'AKI-ND' without further elaboration. The event rate in the validation cohorts is not mentioned. There is no information on the number of predictors that were considered at the start compared to the final number of selected variables, and how these were chosen in the first place.
	Safari et al.	No	Definition of AKI and rhabdomyolysis across the different studies is not provided. There is no information on the number of predictors that were considered at the start compared to the final number of selected variables, and how these were chosen in the first place.
	Silver et al.	No	There is no information on the number of predictors that were considered at the start compared to the final number of selected variables, and how these were chosen in the first place. It is unclear whether the predictors were measured/assessed in the same way in the validation vs the development studies. The number of AKI events in the external validation studies is not available for all studies (missing for Liu-2014, Tziakas-2014). The AKI definition is not provided for several of the external validation studies (missing for Sgura-2010, Tziakas-2014 and Liu-2014) The studies by Liu et al. (2014) and Tziakas et al (2013, including 5571 patients) are not included in the reference list.

	Wilson et al.	No	There is no information on how the choice for predictors was made by the authors of the primary papers. It is unclear how certain predictors were measured. E.g. in the studies that use renal factors as a predictor it is not elaborated what efforts were done to distinguish between AKI and CKD and how the use of a parameter, both as a predictor and as an outcome measure will influence the model's usability.
3.3 Were all relevant study results collected for use in the synthesis?	Allen et al.	Yes	
	Caragata et al.	Yes	
	Hodgson et al.	Yes	
	Huang et al.	Yes	
	Huen et al.	Yes	
	Safari et al.	No	No information on discrimination or calibration of the RPMs is available.
	Silver et al.	Yes	
	Wilson et al.	Yes	
3.4 Was risk of bias (or methodological quality) formally assessed using appropriate criteria?	Allen et al.	Yes	The reviewers used the CHARMS checklist and TRIPOD statement to formulate questions relating to methodological quality.
	Caragata et al.	No	No ROB assessment has been carried out.
	Hodgson et al.	Yes	ROB was assessed by calculating a TRIPOD score and by using the PROBAST tool.
	Huang et al.	No	No ROB assessment has been done.
	Huen et al.	Probably yes	Authors formulated criteria to assess ROB and these criteria seem appropriate.
	Safari et al.	No	Authors state that the methodological quality of the included studies was evaluated using guidelines suggested by the Agency for Healthcare Research and Quality's Methods Guide for Effectiveness and Comparative Effectiveness Reviews. This quality evaluation is not available to the reader.
	Silver et al.	Yes	The criteria used for assessing methodological quality are predefined and seem appropriate.
	Wilson et al.	Yes	Quality assessment criteria were based on the TRIPOD statement. An overview of the assessment is available, but the individual assessment of each included study is not available.

3.5 Were efforts made to minimize error in risk of bias assessment?	Allen et al.	Probably yes	It is not explicitly stated that the ROB assessment was done by two independent reviewers but since study selection and data extraction was done by two independent reviewers, ROB assessment was likely also done by two independent reviewers.
	Caragata et al.	No	No ROB assessment has been done.
	Hodgson et al.	Yes	The ROB assessment was performed by two authors independently.
	Huang et al.	No	No ROB assessment has been done.
	Huen et al.	Probably no	There is no statement on how the ROB assessment was done.
	Safari et al.	Probably yes	Although there is no explicit statement that two reviewers independently assessed methodological quality, there is mention of an 'interrater variability in the assessment' in the text, suggesting multiple reviewers performed the assessment.
	Silver et al.	Yes	The footnote of table 4 describes that two reviewers performed the ROB assessment.
	Wilson et al.	No information	It is not mentioned whether the quality assessment is conducted by two independent reviewers.
Domain 4: Synthesis and findings	Review	Rating	Reasoning
4.1 Did the synthesis include all studies that it should?	Allen et al.	Probably no	Among the 70 models for which performance on prediction of CA-AKI was reported, reports for 39 models included the model's c statistic and 36 of these provided enough information to estimate its variance. In figure 3 only these 36 reports are included in the forest plot. In figure 4 only 6 of the 11 studies of RPMs for CA-AKI with RRT need were included.<br>Several studies are thus excluded from the quantitative analysis and it is unclear whether the authors did all the necessary to contact the authors of the primary studies in an attempt to collect the necessary information that would allow inclusion in the synthesis. Not all studies were discussed in the qualitative analysis.
	Caragata et al.	Yes	
	Hodgson et al.	No	Two external validation studies are not included in the synthesis.
	Huang et al.	Yes	
	Huen et al.	No	The external validation studies are not included in the synthesis.

	Safari et al.	Yes	
	Silver et al.	No	The external validation studies are not included in the synthesis.
	Wilson et al.	Yes	
4.2 Were all pre-defined analyses reported or departures explained?	Allen et al.	No	The reason behind the grouping of the included studies is not clearly defined and this decision seems to be made post hoc. Authors do not explain why they made a distinction between AKI and AKI-RRT and grouped patients accordingly.<br>Authors mention a predefined protocol but this is not available to the reader.
	Caragata et al.	No information	A predefined protocol is not available.
	Hodgson et al.	Yes	
	Huang et al.	No information	A predefined protocol is not available.
	Huen et al.	No information	A predefined protocol is not available.
	Safari et al.	No information	A predefined protocol is not available.
	Silver et al.	No information	A predefined protocol is not available.
	Wilson et al.	Probably yes	
4.3 Was the synthesis appropriate given the nature and similarity in the research questions, study designs and outcomes across included studies?	Allen et al.	Probably no	A meta-analysis of the performance of the external validation studies of several RPMs is performed (figure 5). However, not all the external validation studies use the same outcome definition as the original development study which makes such a quantitative analysis inappropriate. Overall, a purely qualitative analysis seems more appropriate. The quantitative analysis of RPMs for CA-AKI with RRT need in figure 4 includes 6 of the 11 studies evaluating/developing RPMs in this context. Since clinical practice on when to start dialysis for CA-AKI differs widely across centers, heterogeneity in clinical characteristics across these studies is likely but insufficient information is provided to judge this.

	Caragata et al.	Probably no	The mean AUROC of the predictive models was calculated. Such a quantitative synthesis does not seem appropriate because of the heterogeneity across the RPMs.
	Hodgson et al.	Yes	A quantitative synthesis is not feasible because of heterogeneity across studies (mainly because of the variation in AKI definition).
	Huang et al.	Yes	A quantitative synthesis is not feasible because of heterogeneity across studies (mainly because of the variation in AKI definition).
	Huen et al.	Yes	A quantitative synthesis is not feasible because of heterogeneity across studies (mainly because of the variation in AKI definition).
	Safari et al.	Probably no	Combining heterogeneous studies both on individual risk prediction and risk factor finding (dipstick) across completely different settings seems inappropriate. The clinical characteristics of the included studies are too heterogenous to perform a meaningful synthesis.
	Silver et al.	Yes	A narrative synthesis was conducted since there is too much heterogeneity in the reviewed studies to conduct a quantitative meta-analysis.
	Wilson et al.	Yes	A narrative synthesis was conducted since there is too much heterogeneity in the reviewed studies to conduct a quantitative meta-analysis.
4.4 Was between-study variation (heterogeneity) minimal or addressed in the synthesis?	Allen et al.	No	Heterogeneity is assessed across the studies included in the meta-analysis of the external validation studies in figure 5, and considered high, but authors do not explore this item further except with the statement that “this is probably explained by the CA-AKI definition of the external validation study”. Authors state that there is no heterogeneity across the studies analyzed in figure 4 (RPMs for CA-AKI with RRT). However, several studies are omitted in this analysis because of missing data. It is unclear whether authors did all the necessary to retrieve information from the original authors of the primary studies.
	Caragata et al.	No	The authors performed a partial quantitative synthesis by calculating the mean AUROC. There is no consideration for potential heterogeneity across the studies.
	Hodgson et al.	Yes	
	Huang et al.	Yes	The authors do not clearly address the heterogeneity of the studies, but they performed a narrative synthesis and chose not to combine the study results.

	Huen et al.	Yes	A narrative synthesis was conducted. It was not specifically mentioned that the heterogeneity of studies led to this decision.
	Safari et al.	Yes	The authors do not clearly address the heterogeneity of the studies, but they performed a narrative synthesis and chose not to combine the study results.
	Silver et al.	Yes	The heterogeneity of the studies was assessed by mentioning that the studies were too heterogenous to perform a meta-analysis.
	Wilson et al.	Yes	The authors do not clearly address the heterogeneity of the studies, but they performed a narrative synthesis and chose not to combine the study results.
4.5 Were the findings robust, e.g. as demonstrated through funnel plot or sensitivity analyses?	Allen et al.	No	A funnel plot was performed of c-statistics for RPMs for CA-AKI (supplementary material). This funnel plot suggested the presence of small study effects, with a correlation between studies of smaller cohorts reporting higher c-statistics. The presentation of the findings of the qualitative analysis is confusing. There is no explanation as to why authors grouped patients according to severity of AKI. Results might have been different looking at the group as a whole. Since there is no prespecified protocol available to the reader it is difficult to exclude the possibility that these decisions were made after the search and retrieval of citations.
	Caragata et al.	No	The results' section is organized in paragraphs on e.g. liver disease severity, liver disease etiology and other predictors and does not focus on assessing and comparing the quality of the included primary studies
	Hodgson et al.	Yes	
	Huang et al.	No	Comparing the included primary studies by focusing on their methodological quality and including a ROB assessment would have allowed a more critical appraisal of the RPMs.
	Huen et al.	Yes	
	Safari et al.	No	The conclusion is made upon the combination of studies on RPMs and studies on urine dipstick. We believe that a synthesis of RPMs only could lead to a different conclusion.
	Silver et al.	Yes	
	Wilson et al.	Yes	

4.6 Were biases in primary studies minimal or addressed in the synthesis?	Allen et al.	No	There is only a small paragraph on the assessment of methodological quality in those studies that provide tools for individual risk prediction. Authors did not perform a formal ROB assessment.
	Caragata et al.	No	No ROB assessment was conducted.
	Hodgson et al.	Yes	
	Huang et al.	No	No ROB assessment was conducted.
	Huen et al.	No	A ROB assessment has been performed for the seven development studies. This assessment was not thoroughly addressed in the synthesis.
	Safari et al.	No	A formal ROB assessment of the included primary studies was not performed. Authors state the following: “ Quality of the studies was evaluated using guidelines suggested by the agency for Healthcare Research and Quality’s Methods Guide for Effectiveness and Comparative Effectiveness Reviews. Studies were enrolled only if they had a good or fair quality rating. Scoring each article was done based on its design, selection bias, performance, and finale outcome report”. The result of this assessment, also leading to exclusion of 5 citations, is not accessible to the reader.
	Silver et al.	Yes	
	Wilson et al.	Yes	

Abbreviations: AKI = acute kidney injury; AKI-D = acute kidney injury with dialysis; AKI-ND = acute kidney injury without dialysis; ARF = acute renal failure; AUROC = area under the receiver operating characteristics; CA-AKI = contrast associated acute kidney injury; CHARMS = critical appraisal and data extraction for systematic reviews of prediction modelling studies; MESS = mangled extremity severity score; MOOSE = meta-analysis of observational studies in epidemiology; PROBAST = prediction model risk of bias assessment tool; ROB = risk of bias; ROBIS = a risk of bias assessment tool for systematic reviews; RPM = risk prediction model; RPMs = risk prediction models; RRT = renal replacement therapy; TRIPOD = transparent reporting of a multivariable prediction model for individual prognosis or diagnosis.
