# Supplementary material for: Risk prediction models for acute kidney injury in adults: An overview of systematic reviews
Source: PLoS One. 2021 Apr 1;16(4):e0248899. doi: 10.1371/journal.pone.0248899 (PMC8016311; doi:10.1371/journal.pone.0248899)
Supplement: S3 Table — (PDF) [file pone.0248899.s004.pdf]

Supplementary material: AKI definitions used across the included risk prediction models (RPMs), number of participants included in the RPMs and number of AKI events across the RPMs.

Author	AKI definitions	Included participants*	AKI events**
Allen et al.***	1. ≥ 0,5 mg/dl or ≥ 25% increase in sCr within 48h (Chou-2016, Barbieri-2015, Tziakas-2013, Chong-2012, Ghani-2009, Tziakias-2014; Sgura-2010)	1. 539 + 2.308 + 688 + 770 + 347 + 2.689 + 891	1. 55 + 281 + 78 + 88 + 18 + NS + NS
	2. ≥ 0,3 mg/dl increase in sCr within 48h or ≥ 50% increase within 7d or new onset dialysis within 7d (Brown-2015)	2. 143.538	2. 17.566
	3. ≥ 0,3 mg/dl or ≥ 50% increase in sCr within 30d (Inohara-2015)	3. 5.936	3. 358 (NS for IV cohort)
	4. ≥ 0,5 mg/dl increase in sCr within 48-72h (Liu-2015, Liu-2015, Tan 2012)	4. 728 + 1.020 + 1.140	4. 15 (NS for IV cohort) + 39 + 55
	5. ≥ 0,3 mg/dl increase or ≥ 50% increase in sCr or new onset of dialysis within index hospitalization (Tsai-2014, Inohara-2016)	5. 947.012 + 11.041	5. 72.542 + NS
	6. ≥ 0,5 mg/dl or ≥ 25% increase in sCr within 5d, dialysis at one year (Chen-2014)	6. 2.500	6. 418
	7. ≥ 0,5 mg/dl increase in sCr within 1w or new onset of dialysis during index hospitalization (Gurm-2013)	7. 68.573	7. 1.748
	8. ≥ 0,5 mg/dl increase in sCr within 5d (Maioli-2010)	8. 1.720	8. 168
	9. ≥ 2 mg/dl or ≥ 50% increase in sCr or new onset dialysis during index hospitalization (Brown-2008)	9. 11.141	9. 83
	10. ≥ 0,5 mg/dl or ≥ 25% increase in sCr within 72h (Ji-2015, Gao-2014, Ivanes-2014, Ando-2013, Uyarel-2009)	10. 805 + 3.945 + 322 + 481 + 2.521	10. 96 + 177 + NS + NS + 630
	11. ≥ 0,5 mg/dl or ≥ 25% increase in sCr within 1w (Abe-2014, Sato-2015)	11. 1.954 + 2.198	11. NS + NS
	12. ≥ 0,5 mg/dl or ≥ 25% increase in sCr within 48-72h (Fu-2013)	12. 945	12. 151
	13. ≥ 0,5 mg/dl increase in sCr within 72h (Raposeiras-Roubin-2013, Marenzi-2004)	13. 940 + 208	13. 54 + 40
	14. ≥ 0,5 mg/dl increase in sCr within index hospitalization (Liu-2012, Laskey-2007)	14. 277 + 3.179	14. 25 + 48
	15. ≥ 0,5 mg/dl increase in sCr or oligo/anuria within 72h (Nyman-2008)	15. 391	15. 72

	16. ≥ 1 mg/dl increase in sCr within index hospitalization (Bartholomew-2004)<br>17. ≥ 0,5 mg/dl or ≥ 25% increase in sCr within 48h or new onset dialysis during index hospitalization (Mehran-2004)<br>18. New onset dialysis during hospitalization (Freeman-2002)<br>19. ≥ 1 mg/dl or ≥ 25% or ≥ 50% or ≥ 100% increase in sCr or ≥5 mg/dl value of sCr or new onset dialysis within 5d (McCullough-1997)<br>20. ≥ 0,5 mg/dl increase in sCr within 48h or ≥ 25% increase if baseline sCr > 1 mg/dl (Abellas-Sequeiros-2016)<br>21. ≥ 0,3 mg/dl or ≥ 0,5 mg/dl or ≥ 50% increase in sCr within 48-72h (Liu-2014)<br>22. ≥ 0,3 mg/dl or ≥ 50% increase in sCr within 48 (Morabito-2012)<br>23. ≥ 0,5 mg/dl or ≥ 25% increase in sCr within 72h or ≥0,3 mg/dl within 48h (Koo-2013)<br>24. ≥ 0,5 mg/dl or ≥ 1 mg/dl increase in sCr within index hospitalization (Skelding-2007)	16. 20.479<br>17. 8.357<br>18. 14.624<br>19. 3.695<br>20. 120<br>21. 251<br>22. 491<br>23. 735<br>24. 3.213	16. 413<br>17. 1.115<br>18. 82<br>19. 282 (NS for IV cohort)<br>20. NS<br>21. NS<br>22. NS<br>23. NS<br>24. NS
Caragata et al.	1. RRT need postoperatively (Contreras-2002 (early: ≤ 7d postoperatively, late: ≥ 7d postoperatively), Sanchez-2004)<br>2. RRT need ≤ 7d postoperatively (Kim-2014)<br>3. sCr > 132 µmol/l and RRT need < 10d postoperatively (Rueggeberg-2008)<br>4. sCr 133 µmol/l with an increase of 50% and/or RRT need < 7d postoperatively (Xu-2010)<br>5. AKIN criteria < 48h postop (Portal-2010)<br>6. ≥ 26.5 µmol/l or ≥ 50% increase in sCr from baseline within 72h (Hilmi-2015)	1. 260 + (724 + 142)<br>2. 157<br>3. (71 + 167)<br>4. (102 + 44)<br>5. 80<br>6. 424	1. (11% early + 6% late) + (12% + NS for IV cohort)<br>2. 26.8%<br>3. (18.3% + NS for IV cohort)<br>4. (32.4% + NS for IV cohort)<br>5. 37.5%<br>6. 52%
Hodgson et al.	1. Reduction of eGFR to ≤ 50ml/min < 7d post-op (Kheterpal-2007)<br>2. ≥ 177 µmol/l increase in sCr from preoperative value or RRT < 30d (Kheterpal-2009)<br>3. KDIGO sCr changes < 7d (Bell-2015, Forni-2013)	1. 15.102<br>2. 75.952<br>3. 10.615 + 3.523	1. 121<br>2. 762<br>3. 967 + 155

	4. ≥ 44 µmol/l increase in sCr if baseline sCr ≤ 168 µmol/l, ≥ 88 µmol/l if baseline sCr 177-433 µmol/l and ≥ 133 µmol/l if baseline sCr ≥ 442 µmol/l (Drawz-2008)<br>5. ≥ 2 sCr results ≥ 150% of baseline (risk), or ≥ 200% of baseline (injury) within 30d post admission (Matheny-2010)<br>6. AKI and AKI stage 2/3 within 72h according to KDIGO sCr (Bedford-2016)<br>7. AKI within 24h according to KDIGO sCr (Koyner-2016)<br>8. ≥ 26,5 µmol/l increase in sCr during admission (Forman-2004, Breidthardt-2011)<br>9. ≥ 26,5 µmol/l or ≥ 50% increase in sCr within 48h (Wang-2013)<br>NS for EV reports (Xing-2012, Hodgson-2017)	4. 180 cases + 360 controls<br>5. 26.107<br>6. 11.655<br>7. 202.961<br>8. 1.004 + 657<br>9. 1.709<br>NS for EV reports	4. 180 cases + 360 controls<br>5. 2.078<br>6. 361 (NS for IV cohort)<br>7. 17.541<br>8. 271 + 136<br>9. 550<br>NS for EV reports
Huang et al.	1. KDIGO sCr within 1w after study enrollment (Malhotra-2017)<br>2. KDIGO sCr within 1w after ICU admission (Flechet-2017)<br>3. AKIN sCr and/or UO during ICU hospitalization (Chiofolo-2019)<br>4. KDIGO sCr and/or UO within 1w after ICU enrollment (Deng-2017)<br>5. KDIGO sCr within 72h after ICU admission (Zimmerman-2019)	1. (573 + 144 + 1.300)<br>2. (2.123 +2.367)<br>3. (4.572 + 1.958)<br>4. 1.084<br>5. (19.160 + 4.790)	1. (22% + 24% + 45%)<br>2. (27.7% + 29.2%)<br>3. (30% + 30%)<br>4. 30.1%<br>5. (16.5% + 16.5%)
Huen et al.	1. RRT need within 30d postoperatively (Chertow-1997)<br>2. RRT need (Thakar-2005, Mehta-2006, Wijeysundera-2007)<br>3. Postoperative sCr > 2 mg/dl and an increase more than 0,7 mg/dl from baseline or renal failure (RRT need or evidence of renal failure on autopsy) (Aronson-2007)<br>4. Increase of sCr > 2 mg/dl in patients with baseline < 1,5 mg/dl or 50% increase if baseline 1,5-3 mg/dl within 7d postoperatively (Palomba-2007)<br>5. eGFR < 30 ml/min postoperatively (Brown-2007)<br>NS for EV reports (Fortescue-2000, Thakar-2003, Eriksen-2003, Di Bella-2007, Candela-Toha-2008, Heise-2010, Englberger-2010 and Knapik-2008)	1. (42.773 + 3.795)<br>2. (15.838 + 15.839) + (449.524 + 86.009) + (10.751 + 2.566 + 6.814)<br>3. (2.381 + 2.420)<br>4. (603 + 215)<br>5. 8.363<br>EV reports: 8.797 + 22.589 + 2.037 + 1.642 +	1. (1.1% + NS for IV cohort)<br>2. (1.7% + NS for IV cohort) + (1.4% + 1.6%) + (1.3% + NS for IV cohort + NS for EV cohort)<br>3. (4.8% + NS for IV cohort)<br>4. (11% + NS for IV cohort)<br>5. 3%<br>NS for EV reports

		1.780 + 3.508 + 12.096 + 1.421	
Safari et al.	NS	191<br>1.468<br>55<br>79<br>2.962<br>207	9<br>94<br>5<br>8<br>200<br>9
Silver et al.	1. ≥ 1 mg/dl increase in sCr within 48h (Bartholemew-2004)<br>2. > 0,5 mg/dl or 25% increase in sCr within 5d (Chen-2014)<br>3. > 0,5 mg/dl or 25% increase in sCr within 48-72h (Fu-2013)<br>4. > 0,5 mg/dl increase in sCr within 48h (Ghani-2009, Marenzi-2004)<br>5. > 0,5 mg/dl or 25% increase in sCr within 72h (Gao-2014)<br>6. > 0,5 mg/dl increase in sCr within 7d (Gurm-2013)<br>7. > 0,5 mg/dl increase in sCr within 48-72h (Liu-2015)<br>8. > 0,5 mg/dl increase in sCr within 5d (Maioli-2010)<br>9. > 0,5 mg/dl or 25% increase in sCr within 48h (Mehran-2004, Tziakas-2013, Victor-2014)<br><br>NS for EV reports (Tziakas-2014, Liu-2014§, Sgura-2010, Tziakas-2013§)	1. 20.478<br>2. 2.500<br>3. 945<br>4. 347 + 218<br>5. 3.945<br>6. 68.573<br>7. 728<br>8. 1.783<br>9. 8.357 + 688 + 1.200<br><br>EV reports: 2.689 + 728 + 891 + 5.571	1. 413 + NS<br>2. 418<br>3. 151<br>4. 18 + 166<br>5. 177<br>6. 1.748<br>7. 15 (NS for IV cohort)<br>8. 168<br>9. 1.115 + 75 + 113 + NS + NS
Wilson et al.	1. > 2 mg/dl increase in sCr or RRT need within 30d (Kheterpal-2009)<br>2. RRT need within 1w (Kim-2014)<br>3. sCr > 132 µmol/l and RRT need (Rueggeberg-2008)<br>4. RRT need (Sanchez-2004)<br>5. 0,3 mg/dl increase or 1,5 times baseline increase within 48h or oliguria < 0,5 ml/kg/h for at least 6h (Slankamenac-2009, Slankamenac-2013)	1. 75.952<br>2. 157<br>3. 238<br>4. 866<br>5. 569 + 549	1. 762<br>2. 42<br>3. 36<br>4. 107<br>5. 86 + 82

Abbreviations: AKI = Acute Kidney Injury; AKIN = Acute Kidney Injury Network; d = days; eGFR = estimated glomerular filtration rate; EV = external validation; h = hours; ICU = Intensive Care Unit; IV = internal validation; KDIGO = Kidney Disease: Improving Global Outcomes; NS = Not Stated; sCr = serum creatinine; RRT = renal replacement therapy; UO = urinary output; w = week.

* Total number of participants included in both the development cohort and the internal and external validation cohort, if applicable. The "+" symbol is either used to distinguish between different studies using the same definition or facilitates the interpretation of the number of AKI events if only percentages are used to indicate the latter. Thus in those RPMs were only event percentages are provided, we chose to formulate separate numbers for both the development and the internal and external validation cohorts within the same study (if applicable), instead of using a total number and made this graphically clear by using brackets.

** Number of AKI events including development cohort, internal validation cohort and external validation cohort (if applicable). If the number is stated as a percentage, the different percentages across respectively development cohort, internal validation cohort and external validation cohort (if applicable) are provided.

*** For Allen et al. the total number of included patients refers to the number of patients included in the 30 models that provided sufficient information to obtain individual risk estimates. Authors included 75 reports in the review but only provided these numbers for 30 models. The AKI definitions in the table are those that were extracted from these 30 models.

§ The reference of the study by Liu et al. 2014 and Tziakas et al. 2013 (including 5.571 participants) could not be retrieved from the reference list provided by the authors.
