# Supplementary material for: Risk prediction models for acute kidney injury in adults: An overview of systematic reviews
Source: PLoS One. 2021 Apr 1;16(4):e0248899. doi: 10.1371/journal.pone.0248899 (PMC8016311; doi:10.1371/journal.pone.0248899)
Supplement: S1 File — (PDF) [file pone.0248899.s005.pdf]

Overview of systematic reviews of risk prediction models in AKI

MEDLINE – first search performed on 17/01/2020: 2003-current

1. (validat* or predict*).ti.
2. ((scor* or risk* or predict* or prognostic*) adj1 (system* or model* or equation* or tool*)).tw.
3. ((risk* or predict* or prognostic*) adj1 scor*).tw.
4. receiver operating characteristic*.tw.
5. c statistic.tw.
6. calibration.tw.
7. Proportional Hazards Models/
8. ROC Curve/
9. exp Risk Assessment/
10. exp Acute Kidney Injury/
11. (acute kidney failure or acute renal failure).tw.
12. (acute kidney injur$ or acute renal injur$).tw.
13. (acute kidney insufficie$ or acute renal insufficie$).tw.
14. acute tubular necrosis.tw.
15. (ARI or AKI or ARF or AKF or ATN).tw.
16. contrast induced nephropath*.tw.
17. contrast-induced nephropath*.tw.
18. ((radiocontrast* or contrast*) adj4 nephrotoxic*).tw.
19. (contrast* adj4 nephropath*).tw.
20. 1 or 2 or 3 or 4 or 5 or 6 or 7 or 8 or 9
21. 10 or 11 or 12 or 13 or 14 or 15 or 16 or 17 or 18 or 19
22. 20 and 21
23. animals/ not (humans/ and animals/)
24. 22 not 23
25. limit 24 to english language
26. limit 25 to yr="2003 -Current"

MEDLINE - updated search performed on 10/08/2020: 17/01/2020 - current

1. (validat* or predict*).ti.
2. ((scor* or risk* or predict* or prognostic*) adj1 (system* or model* or equation* or tool*)).tw.
3. ((risk* or predict* or prognostic*) adj1 scor*).tw.
4. receiver operating characteristic*.tw.
5. c statistic.tw.

6. calibration.tw.
7. Proportional Hazards Models/
8. ROC Curve/
9. exp Risk Assessment/
10. exp Acute Kidney Injury/
11. (acute kidney failure or acute renal failure).tw.
12. (acute kidney injur$ or acute renal injur$).tw.
13. (acute kidney insufficie$ or acute renal insufficie$).tw.
14. acute tubular necrosis.tw.
15. (ARI or AKI or ARF or AKF or ATN).tw.
16. contrast induced nephropath*.tw.
17. contrast-induced nephropath*.tw.
18. ((radiocontrast* or contrast*) adj4 nephrotoxic*).tw.
19. (contrast* adj4 nephropath*).tw.
20. 1 or 2 or 3 or 4 or 5 or 6 or 7 or 8 or 9
21. 10 or 11 or 12 or 13 or 14 or 15 or 16 or 17 or 18 or 19
22. 20 and 21
23. animals/ not (humans/ and animals/)
24. 22 not 23
25. limit 24 to english language
26. limit 25 to dt=20200117-20200817

EMBASE – first search performed on 20/01/2020: 2003-current

No.	Query	Results	Date
1	validat*:ti OR predict*:ti	540263	20/jan/20
2	((scor* OR risk* OR predict* OR prognostic*) NEAR/1 (system* OR model* OR equation* OR tool*)):ti,ab,kw	182217	20/jan/20
3	((risk* OR predict* OR prognostic*) NEAR/1 scor*):ti,ab,kw	56194	20/jan/20
4	'receiver operating characteristic*':ti,ab,kw	83205	20/jan/20
5	'c statistic':ti,ab,kw	7969	20/jan/20
6	calibration:ti,ab,kw	86003	20/jan/20
7	'proportional hazards model'/de	86484	20/jan/20
8	'receiver operating characteristic'/de	119061	20/jan/20
9	'risk assessment'/exp	536490	20/jan/20
10	'acute kidney failure'/de	84736	20/jan/20
11	'acute kidney tubule necrosis'/de	4770	20/jan/20
12	'acute kidney failure':ti,ab,kw OR 'acute renal failure':ti,ab,kw	34220	20/jan/20
13	'acute kidney injur*':ti,ab,kw OR 'acute renal injur*':ti,ab,kw	36092	20/jan/20
14	'acute kidney insufficie*':ti,ab,kw OR 'acute renal insufficie*':ti,ab,kw	2154	20/jan/20
15	'acute tubular necrosis':ti,ab,kw	4697	20/jan/20
16	ari:ti,ab,kw OR aki:ti,ab,kw OR arf:ti,ab,kw OR akf:ti,ab,kw OR atn:ti,ab,kw	40935	20/jan/20
17	'contrast induced nephropath*':ti,ab,kw	3564	20/jan/20
18	((radiocontrast* OR contrast*) NEAR/4 nephrotoxic*):ti,ab,kw	1077	20/jan/20

19	(contrast* NEAR/4 nephropath*):ti,ab,kw	4554	20/jan/20
20	#1 OR #2 OR #3 OR #4 OR #5 OR #6 OR #7 OR #8 OR #9	1428590	20/jan/20
21	#10 OR #11 OR #12 OR #13 OR #14 OR #15 OR #16 OR #17 OR #18 OR #19	124205	20/jan/20
22	#20 AND #21	11279	20/jan/20
23	#22 NOT ([animals]/lim NOT [humans]/lim)	11086	20/jan/20
24	#23 AND [english]/lim	10808	20/jan/20
25	#24 AND [2003-2020]/py	10458	20/jan/20

EMBASE - updated search performed on 17/08/2020: 20/01/2020 – current

No.	Query	Results	Date
1	validat*:ti OR predict*:ti	540263	17/aug/20
2	((scor* OR risk* OR predict* OR prognostic*) NEAR/1 (system* OR model* OR equation* OR tool*)):ti,ab,kw	182217	17/aug/20
3	((risk* OR predict* OR prognostic*) NEAR/1 scor*):ti,ab,kw	56194	17/aug/20
4	'receiver operating characteristic*':ti,ab,kw	83205	17/aug/20
5	'c statistic':ti,ab,kw	7969	17/aug/20
6	calibration:ti,ab,kw	86003	17/aug/20
7	'proportional hazards model'/de	86484	17/aug/20
8	'receiver operating characteristic'/de	119061	17/aug/20
9	'risk assessment'/exp	536490	17/aug/20
10	'acute kidney failure'/de	84736	17/aug/20
11	'acute kidney tubule necrosis'/de	4770	17/aug/20
12	'acute kidney failure':ti,ab,kw OR 'acute renal failure':ti,ab,kw	34220	17/aug/20
13	'acute kidney injur*':ti,ab,kw OR 'acute renal injur*':ti,ab,kw	36092	17/aug/20
14	'acute kidney insufficie*':ti,ab,kw OR 'acute renal insufficie*':ti,ab,kw	2154	17/aug/20
15	'acute tubular necrosis':ti,ab,kw	4697	17/aug/20
16	ari:ti,ab,kw OR aki:ti,ab,kw OR arf:ti,ab,kw OR akf:ti,ab,kw OR atn:ti,ab,kw	40935	17/aug/20
17	'contrast induced nephropath*':ti,ab,kw	3564	17/aug/20
18	((radiocontrast* OR contrast*) NEAR/4 nephrotoxic*):ti,ab,kw	1077	17/aug/20
19	(contrast* NEAR/4 nephropath*):ti,ab,kw	4554	17/aug/20
20	#1 OR #2 OR #3 OR #4 OR #5 OR #6 OR #7 OR #8 OR #9	1428590	17/aug/20
21	#10 OR #11 OR #12 OR #13 OR #14 OR #15 OR #16 OR #17 OR #18 OR #19	124205	17/aug/20
22	#20 AND #21	11279	17/aug/20
23	#22 NOT ([animals]/lim NOT [humans]/lim)	11086	17/aug/20
24	#23 AND [english]/lim	10808	17/aug/20
25	#24 AND [20-1-2020]/sd NOT [2-7-2021]/sd	1112	17/aug/20
